# Anforderung von radiologischer Diagnostik in der Unfallchirurgie mittels mobiler Endgeräte

**DOI:** 10.1007/s00113-024-01410-8

**Published:** 2024-02-01

**Authors:** Konrad F. Fuchs, Fabian Kerwagen, Andreas S. Kunz, Andrés Schulze, Melanie Ullrich, Maximilian Ertl, Fabian Gilbert

**Affiliations:** 1 Klinik und Poliklinik für Unfall‑, Hand‑, Plastische und Wiederherstellungschirurgie, Universitätsklinikum Würzburg, Würzburg, Deutschland; 2 Medizinische Klinik und Poliklinik I, Universitätsklinikum Würzburg, Würzburg, Deutschland; 3 Institut für Diagnostische und Interventionelle Radiologie, Universitätsklinikum Würzburg, Würzburg, Deutschland; 4 Digitalisierungszentrum Präzisions- und Telemedizin, Universitätsklinikum Würzburg, Würzburg, Deutschland; 5 LMU Klinikum Großhadern, Muskuloskelettales Universitätszentrum München, München, Deutschland

**Keywords:** Digitalisierung, Prozessoptimierung, Radiologie, Smartphone, App, Digitalization, Process optimization, Radiology, Smartphone, App

## Abstract

**Hintergrund:**

Ärztliches Personal steht täglich unter hohem zeitlichen Druck. Eine ärztliche Aufgabe ist die Anforderung von radiologischer Diagnostik. Dieser Prozess zeichnet sich durch eine hohe administrative Komplexität und teils enormen zeitlichen Aufwand aus. Maßnahmen, die zugunsten der Versorgung von Patientinnen und Patienten zu einer administrativen Entlastung führen, fehlen bisher.

**Ziel der Arbeit:**

Prozessoptimierung in der Anforderungsstellung von radiologischer Diagnostik. Als „proof of concept“ wurde in der unfallchirurgischen Abteilung am Universitätsklinikum Würzburg (UKW) die Anforderung radiologischer Diagnostik mittels einer Smartphone- und Tablet-basierten Applikation mit Spracheingabe eingeführt.

**Material und Methoden:**

In einer prospektiven Studie wurden der zeitliche Effekt und die zeitliche Effizienz der mobilen, ukw.mobile App-basierten Anforderung (UMBA) im Vergleich zur PC-basierten Anforderung (PCBA) zur Anforderung radiologischer Leistungen analysiert. Ermittelt wurden die Zeit von Indikationsstellung bis zur fertigen Anforderung und die benötigte Zeit für die Anforderungserstellung am Endgerät. Aufgrund der Nichtnormalverteilung der Daten wurde ein Mann-Whitney-U-Test durchgeführt.

**Ergebnisse:**

Die Zeit von der Indikation bis zur fertigen Anforderung konnte durch die mobile Anforderung statistisch signifikant (*p* < 0,05) reduziert werden (PCBA: Mittelwert ± Standardabweichung [SD] 19,57 ± 33,24 min, Median 3,00 min, Interquartilsabstand [IQR] 1,00–30,00 min vs. UMBA: 9,33 ± 13,94 min, 1,00 min, 0,00–20,00 min). Die Zeit für die Anforderung am Endgerät konnte durch die mobile Anforderung ebenfalls statistisch signifikant reduziert werden (PCBA: Mittelwert ± SD 63,77 ± 37,98 s, Median 51,96 s, IQR 41,68–68,93 s vs. UMBA: 25,21 ± 11,18 s, 20,00 s, 17,27–29,00 s).

**Diskussion:**

Das mobile, sprachunterstützte Anforderungsverfahren führt zu einer enormen zeitlichen Entlastung im klinischen Alltag und verdeutlicht das Potenzial einer anwenderorientierten, zielgerichteten Digitalisierung im Gesundheitswesen. In Zukunft soll der Prozess durch eine künstliche Intelligenz unterstützt werden.

**Graphic abstract:**

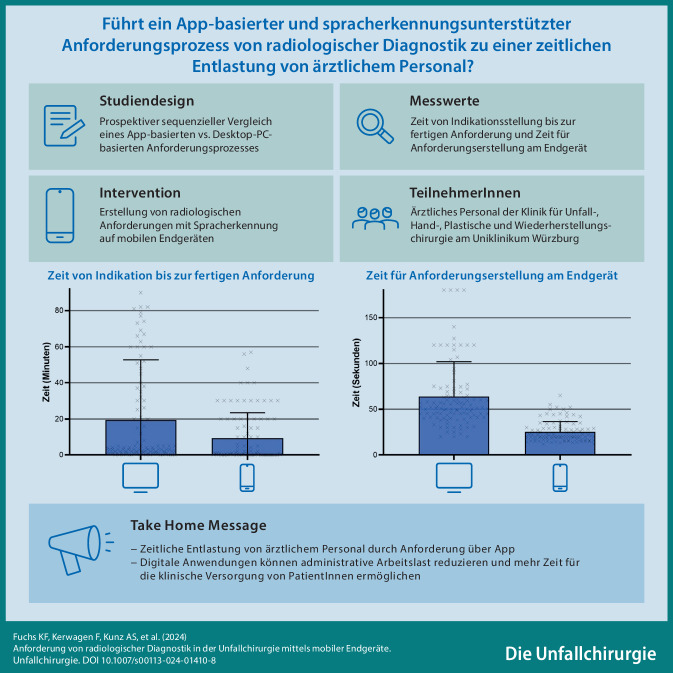

## Abstract (en)

**Background:**

Time is a scarce resource for physicians. One medical task is the request for radiological diagnostics. This process is characterized by high administrative complexity and sometimes considerable time consumption. Measures that lead to an administrative relief in favor of patient care have so far been lacking.

**Aim of the study:**

Process optimization of the request for radiological diagnostics. As a proof of concept the request for radiological diagnostics was conducted using a mobile, smartphone and tablet-based application with dedicated voice recognition software in the Department of Trauma Surgery at the University Hospital of Würzburg (UKW).

**Material and methods:**

In a prospective study, time differences and efficiency of the mobile app-based method (ukw.mobile based Application = UMBA) compared to the PC-based method (PC-based application = PCBA) for requesting radiological services were analyzed. The time from the indications to the completed request and the time required to create the request on the device were documented and assessed. Due to the non-normal distribution of the data, a Mann-Whitney U test was performed.

**Results:**

The time from the indications to the completed request was significantly (*p* < 0.05) reduced using UMBA compared to PCBA (PCBA: mean ± standard difference [SD] 19.57 ± 33.24 min, median 3.00 min, interquartile range [IQR] 1.00–30.00 min vs. UMBA: 9.33 ± 13.94 min, median 1.00 min, IQR 0.00–20.00 min). The time to complete the request on the device was also significantly reduced using UMBA (PCBA: mean ± SD 63.77 ± 37.98 s, median 51.96 s, IQR 41.68–68.93 s vs. UMBA: 25.21 ± 11.18 s, median 20.00 s, IQR 17.27–29.00 s).

**Conclusion:**

The mobile, voice-assisted request process leads to a considerable time reduction in daily clinical routine and illustrates the potential of user-oriented, targeted digitalization in healthcare. In future, the process will be supported by artificial intelligence.

## Hintergrund und Fragestellung

Die Digitalisierung spielt in allen Lebensbereichen eine zunehmende Rolle. Lag die private Internetnutzung 2011 noch bei 76 % der Gesamtbevölkerung, stieg sie im Jahr 2018 auf 87 % [18]. Während im privaten Bereich die Nutzung digitaler Angebote und mobiler Endgeräte stetig zunimmt, bleiben die Potenziale der digitalen Medizin häufig noch ungenutzt. Verglichen mit anderen Industrienationen existieren in Deutschland deutliche Defizite bezüglich der Digitalisierung im Gesundheitswesen [5, 6, 8, 17].

Eine Möglichkeit zur Messung des Digitalisierungsgrades in Krankenhäusern bietet das „Electronic Medical Record Adoption Model“ (EMRAM), welches eine Skala von 0 („es wird kaum digital gearbeitet“) bis 7 („papierloses Krankenhaus“) umfasst [13]. Während der Anteil an Kliniken in EMRAM-Stufe 0 im Jahr 2017 in der Türkei bei ca. 7 % lag, wiesen in Deutschland 38,3 % der Kliniken diesen Digitalisierungsgrad auf [13]. Das bedeutet, dass 2017 ca. 40 % der deutschen Kliniken fast ausschließlich in Papierform arbeiteten.

Auf der anderen Seite sind der zeitliche und finanzielle Druck im deutschen Gesundheitssystem allgegenwärtig. Medizinisches Personal hat, insbesondere in Deutschland, ein hohes Risiko, an einem Burn-out oder an Depressionen zu erkranken [1, 2, 10, 11]. Mögliche Ursachen sind Personalmangel, hohes Aufkommen von Patientinnen und Patienten im ambulanten und stationären Sektor und der konstant hohe Leistungsdruck [7, 14, 20, 21].

Der Markt an Gesundheitsapplikationen nimmt stetig zu, im klinischen Alltag finden diese bisher jedoch kaum Anwendung. Digitalisierung in der Medizin kann bedeuten, dass Arbeitsabläufe effizienter gestaltet werden, medizinisches Personal entlastet wird und der Dokumentationsaufwand sinkt. Es sollte daher das Ziel sein, medizinisches Personal so gut wie möglich durch digitale Lösungen zu unterstützen und die Behandlung von Patientinnen und Patienten zu optimieren.

**Figure 1:**
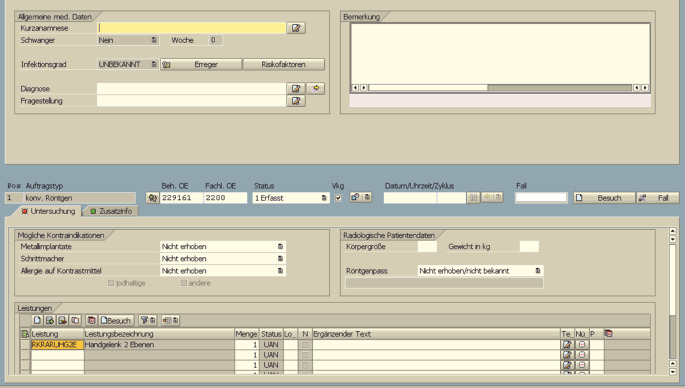

Ein Teil der täglichen klinischen Arbeit, v. a. auf unfallchirurgischen Stationen, besteht darin, radiologische Untersuchungen anzufordern. Am Universitätsklinikum Würzburg (UKW) erfolgt dies am PC mithilfe des Krankenhausinformationssystems (KIS). Bei der PC-basierten Plattform (*i. s. h. med*., Fa. Cerner Health Services Deutschland GmbH, Berlin, Deutschland) wird über ein spezifisches Anforderungsmodul individuell (eineindeutige Fallzuordnung über Patientinnen- bzw. Patientenfallnummer) eine radiologische Anforderung erstellt (Abb. 1). Die Befüllung der Textfelder kann manuell über die Tastatur oder alternativ mittels Spracherkennung (Produkt: *Dragon Medical*, Fa. *Nuance Communications*, Inc., Burlington, MA, USA) erfolgen. Aktuell muss das medizinische Fachpersonal zur Anforderung einer Leistung einen stationären PC aufsuchen, der sich in der Regel im Arztzimmer befindet. Deshalb werden während der morgendlichen Visite zunächst die notwendigen Untersuchungen gesammelt. Die Eingabe am PC bzw. das Anlegen der Untersuchungsanforderung erfolgt dann zu einem späteren Zeitpunkt (z. B. nach Beendigung der Visite). Ferner sind die Anmeldung am PC und die Navigation im KIS (z. B. Suchen der Patientin/des Patienten) z. T. umständlich und zeitaufwendig [15].

Als Alternative zum PC-basierten KIS wurde durch das Servicezentrum Medizin-Informatik (SMI) des Universitätsklinikums Würzburg im Jahr 2016 eine interne IT-Lösung in Form einer Smartphone‑/Tablet-Applikation (ukw.mobile App; Idee und Konzept: Helmut Greger [SMI], technisches Konzept und Entwicklung: Ulrich Trampe [SMI]) entwickelt. Über die App kann auf das *Picture Archiving and Communications System* (Produkt: *PACS*, Fa. Phönix PACS GmbH, Freiburg, Deutschland) sowie auf alle Patientinnen- und Patientendokumente, wie z. B. Laborbefunde, Arztbriefe oder Untersuchungsbefunde, zugegriffen werden. In der Initialversion konnten zunächst lediglich auf Informationen zugegriffen und keine neuen Daten über die App in das KIS eingespeist werden. Im Jahr 2016 wurde die App um die Möglichkeit erweitert, klinische Fotos z. B. zur Wunddokumentation in das PACS zu überspielen [9].

Dem ärztlichen Personal wurde im Jahr 2016 zunächst ein Tabletcomputer (iPad Generation 3–4, Fa. *Apple* Inc., Cupertino, CA, USA) zur Verfügung gestellt. Im Rahmen des durch den bayerischen Staat unterstützten Digitalisierungszentrums für Präzisions- und Telemedizin (DZ.PTM) erfolgte ab 2018 die Ausgabe von iPhones XR (Fa. *Apple* Inc.) an alle ärztlichen Mitarbeiterinnen und Mitarbeiter.

Mit dem Ziel, ärztliches Personal weiter zu entlasten, erfolgte ab 2020 die Erweiterung der ukw.mobile App um die Möglichkeit, radiologische Diagnostik direkt und per Diktat über das mobile Endgerät anzufordern.

## Studiendesign und Untersuchungsmethoden

### Radiologische Anforderungen über die ukw.mobile App

**Figure 2:**
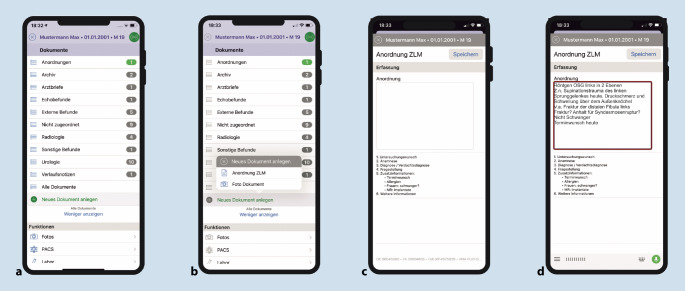

Sobald die klinische Indikation zur Durchführung einer radiologischen Untersuchung durch das ärztliche Personal gestellt wird, kann diese über die ukw.mobile App am Smartphone oder Tablet-PC angefordert werden. Hierzu wird die Patientin/der Patient ausgewählt oder das individuelle Armband mit dem Smartphone eingescannt. Es besteht nun die Möglichkeit, eine Anforderung per Tastatur auf dem Bildschirm oder Spracherkennung einzugeben (Abb. 2). Der fertige Anforderungstext wird anschließend an das neu geschaffene Zentrale Leistungsstellenmanagement (ZLM) übermittelt. Medizinische Fachangestellte übertragen diesen schließlich manuell in die konventionelle PC-Maske und bestätigen die Anforderung. Dieser Zwischenschritt ist aktuell noch notwendig, um eine korrekte Übertragung in das KIS zu ermöglichen. In Zukunft soll dies durch eine künstliche Intelligenz (KI) unterstützt werden. Weitere wichtige Aufgaben, die durch das ZLM realisiert werden, sind die Planung und Terminierung der Untersuchung.

### Datenschutz

Der Schutz der Patientinnen- und Patientendaten stellt eine zwingende Voraussetzung bei der Entwicklung von digitalen Lösungen im Gesundheitswesen dar. Die ukw.mobile App wurde durch das SMI klinikintern unter Berücksichtigung der Datenschutz-Grundverordnung (DSGVO) und des Bayerischen Krankenhausgesetzes (BayKrG) entwickelt. Alle Daten der ukw.mobile App werden auf klinikinternen Serversystemen gespeichert, verbleiben „on premise“ und verlassen somit zu keinem Zeitpunkt das klinikeigene Netzwerk.

### Studiendesign und Endpunkte

Um mögliche Unterschiede des Zeitaufwands zwischen der PC-basierten Anforderung (PCBA) und der ukw.mobile App-basierten Anforderung (UMBA) festzustellen, wurde eine prospektive, sequenzielle Erfassung der Zeiten für die Anforderungserstellung von Röntgenbildern durchgeführt. Es erfolgte zunächst die Erfassung der Zeiten für die PCBA, gefolgt von der Erfassung für die UMBA.

Das ärztliche Personal der Klinik und Poliklinik für Unfall‑, Hand‑, Plastische und Wiederherstellungschirurgie dokumentierte eigenständig in eine vorgefertigte digitale oder ausgedruckte Tabelle folgende Parameter:

- Zeitdauer vom Zeitpunkt der klinischen Indikationsstellung einer radiologischen Untersuchung bis zum Zeitpunkt der fertigen Anforderung am jeweiligen Endgerät (in Minuten),
- Zeitdauer für die Anforderungserstellung am jeweiligen Endgerät (in Sekunden).

### Auswertung und Statistik

Alle vom ärztlichen Personal dokumentierten Werte wurden in einer Excel-Datei (*Excel*, Fa. *Microsoft Corporation*, Redmond, WA, USA) zusammengeführt und schließlich über SPSS Statistics 26 (Fa. *International Business Machines Corporation*, Armonk, NY, USA) ausgewertet. Die grafische Auswertung wurde mit GraphPad Prism 9 (Fa. *GraphPad Software*, Inc., Boston, MA, USA) realisiert. Es erfolgten eine deskriptive Statistik und anschließend ein Kolmogorov-Smirnov-Test auf Normalverteilung. Aufgrund der Nichtnormalverteilung der Daten wurde schließlich ein Mann-Whitney-U-Test durchgeführt. Das Signifikanzniveau wurde auf *p* < 0,05 gesetzt.

## Ergebnisse

**Figure 3:**
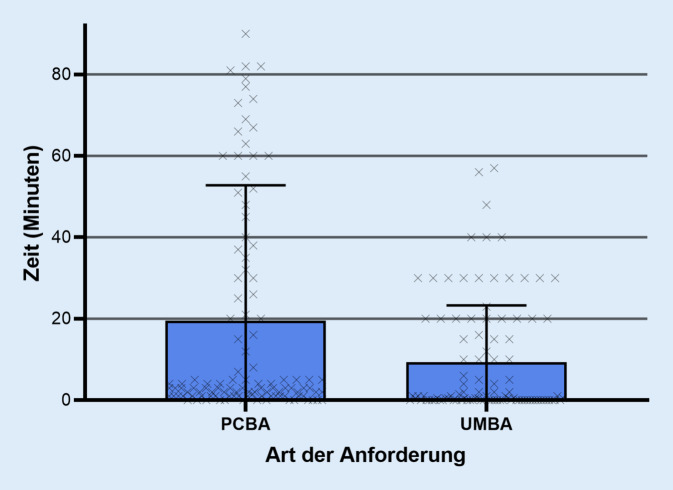

**Figure 4:**
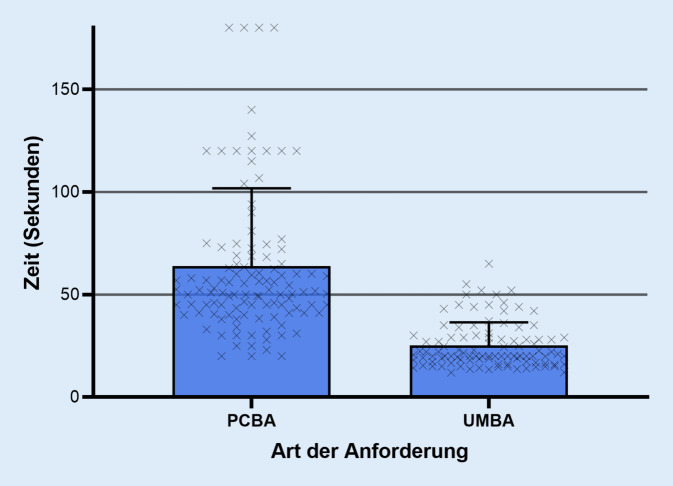

Insgesamt wurden in der Gruppe der PCBA 112 Fälle und in der UMBA 101 Fälle erfasst. Im durchgeführten Mann-Whitney-U-Test Test konnte durch die UMBA eine statistisch signifikante (*p* < 0,001) Reduktion der Zeit von der Indikation bis zur fertigen Anforderung gezeigt werden (PCBA: Mittelwert ± Standardabweichung [SD] 19,57 ± 33,24 min, Median 3,00 min, Interquartilsabstand [IQR] 1,00–30,00 min vs. UMBA: 9,33 ± 13,94 min, 1,00 min, 0,00–20,00 min; Abb. 3). Die Zeit für die UMBA am Endgerät war ebenfalls statistisch signifikant (*p* < 0,001) reduziert (PCBA: Mittelwert ± SD 63,77 ± 37,98 s, Median 51,96 s, IQR 41,68–68,93 s vs. UMBA: 25,21 ± 11,18  s, 20,00 s, 17,27–29 s; Abb. 4).

## Diskussion

In dieser Studie konnte gezeigt werden, dass durch eine spracherkennungsunterstützte mobile Applikation, die für die Erstellung von radiologischen Anforderungen benötigte Zeit im Vergleich zu einer konventionellen Anforderung über einen PC, deutlich reduziert werden konnte.

Digitalisierung kann medizinisches Personal im Alltag erheblich unterstützen, ist häufig jedoch initial mit größeren Investitionen verbunden. Der direkte Nutzen für den klinischen Alltag ist dabei oftmals schwer abzuschätzen und der Markt an Anbietern von entsprechender Software mit adäquatem Datenschutz unübersichtlich. Dabei wird der Umsatz in diesem Bereich voraussichtlich weiterwachsen.

Eine fortschreitende Digitalisierung in der Medizin, v. a. im klinischen Alltag, ist unausweichlich. Deutschland droht, im internationalen Vergleich den Anschluss zu verlieren [13]. Mögliche Gründe hierfür sind ein Investitionsstau, uneinheitliche Konzepte zur Umsetzung der Digitalisierung und ein Mangel an Fachpersonal, das entsprechende Konzepte umsetzen kann. Die Datenlage zum wirklichen Nutzen von Apps in Kliniken ist daher spärlich. Die Bundesregierung hatte am 29.10.2020 das Krankenhauszukunftsgesetz (KHZG) für die Digitalisierung von Krankenhäusern verabschiedet, in dem bis zu 4,3 Mrd. € bereitgestellt wurden, um dies zu ändern [4]. Hier konnten u. a. Fördermittel für Software und krankenhausübergreifende Projekte beantragt werden [22]. Ferner wird in der Digitalisierungsstrategie des Bundesministeriums für Gesundheit das Ziel einer Steigerung der Wirtschaftlichkeit und Effizienz durch nutzenorientierte Anwendungen definiert [23].

Die Möglichkeit, von jedem Bereich in der Klinik auf das KIS zugreifen zu können und zeitgleich Anforderungen zu erstellen, ist ein großer Vorteil gegenüber den konventionellen PC-gebundenen Systemen. Vor allem in dynamischen Fachbereichen, wie der Unfallchirurgie, in denen ärztliches Personal in vielen unterschiedlichen Räumlichkeiten (OP-Bereich, Station, Notaufnahme, Schockraum etc.) agiert, erleichtert dies den klinischen Alltag enorm.

Die am UKW entwickelte Technologie setzt jedoch voraus, dass Kliniken bereits eine digitale Infrastruktur mit mobilen Endgeräten und verlässlicher WLAN-Abdeckung (die nicht in allen Kliniken gegeben ist) besitzen oder sich anschaffen. Dies ist mit hohen Kosten verbunden. Eine Möglichkeit, einen Teil der hohen Anschaffungskosten zu umgehen, wäre, dass das Personal die App auf dem privaten Smartphone installieren kann. Am UKW wurde sich jedoch aus datenschutzrechtlichen Gründen explizit dagegen entschieden.

Technische Neuerungen benötigen ferner eine adäquate Einweisung. Personen, die nicht im Zeitalter der Digitalisierung aufgewachsen sind, benötigen häufiger länger, um neue Technologien zu akzeptieren und anzuwenden [3, 16, 19]. In einer bereits veröffentlichten Studie konnte bei der UMBA eine statistisch signifikant höhere Akzeptanz im Vergleich zur PCBA festgestellt werden [12]. Es ist jedoch erforderlich, das Personal im Umgang mit neuartigen Applikationen, wie der UMBA, regelmäßig zu schulen. Neue Technologien, wie die Implementierung von Smartphones und Tablet-PC, könnten zusätzlich die Attraktivität von Gesundheitseinrichtungen als moderne Arbeitgeber steigern.

## Limitationen

Die hier vorgestellten Daten wurden an einem einzigen Haus der Maximalversorgung erhoben und liefern lediglich eine Aussage über den zeitlichen Nutzen der vorgestellten Applikation. Die Komplexität der Krankheitsbilder und Anamnese, welche erheblich differieren kann, wurde nicht dokumentiert. Solche Details sind im klinischen Betrieb nicht adäquat quantifizierbar und wurden in dieser Untersuchung daher nicht erfasst. Dies spiegelt sich u. a. in der größeren Anzahl an statistischen Ausreißern in der Gruppe der PCBA wider, die sich dadurch erklären lassen, dass die benötigte Zeit für eine PCBA schon prinzipiell länger und dieses Prozedere somit anfälliger für unvorhersehbare Störungen ist. Ob bei der PCBA und UMBA die Spracherkennung genutzt wurde oder nicht, wurde ebenfalls nicht dokumentiert. Es ist daher eine weiterführende Studie mit konstruierten Fällen unter Laborbedingungen in Planung, in der die Usability ausgewertet und der zeitliche Nutzen der UMBA präzisiert werden können. Der Vorteil der vorliegenden Studie ist insbesondere, dass sie im Klinikalltag und im Live-Betrieb durchgeführt wurde, und es zeichnet sich auch ohne Berücksichtigung der oben genannten Limitationen ab, dass der bei der Benutzung der UMBA festgestellte Zeitgewinn sich beim ärztlichen Personal auch im klinischen Alltag manifestiert.

Im aktuellen Prozess der UMBA wird die Arbeit für das Ausfüllen der SAP-Maske an nichtärztliches Personal verlagert. Es ist somit zunächst zusätzliches Personal erforderlich. Auch dies ist mit Kosten verbunden und bietet potenziell die Möglichkeit von Übertragungsfehlern. Aktuell wird im Rahmen des DZ.PTM an Lösungen gearbeitet, um diesen Zwischenschritt durch eine KI zu ersetzen. Durch die Implementierung einer KI in den Anforderungsprozess können künftig zusätzliche Ziele erreicht werden, wie die Präzisierung der radiologischen Diagnostik in der personalisierten Medizin bzw. die Reduktion unnötiger Diagnostik. Eine KI muss jedoch möglichst fehlerfrei und zuverlässig arbeiten, bevor sie im klinischen Alltag Anwendung finden kann.

## Ausblick

Mobile Endgeräte bieten das Potenzial, medizinisches Personal zeitlich zu entlasten. Am UKW ist es das Ziel, die ukw.mobile App auszuweiten, damit andere Anforderungen, wie z. B. Konsile, Laboruntersuchungen etc., aber auch Arztbriefe ebenfalls über mobile Endgeräte angefordert/angelegt werden können.

Es erscheint plausibel, dass der klassische PC im Arbeitsalltag an Gesundheitseinrichtungen in Deutschland weiter an Bedeutung verlieren wird. Die Nutzung von mobilen Endgeräten und optimierten Applikationen kann wichtige Zeit bei administrativen Tätigkeiten einsparen, damit mehr Zeit für die Betreuung von Patientinnen und Patienten bleibt.

## Fazit für die Praxis

- Die Implementierung einer mobilen App mit Spracherkennung zur Anforderung von radiologischer Diagnostik kann ärztliches Personal zeitlich erheblich entlasten.
- Eine Ausweitung des Angebots an mobil anforderbaren Leistungen über die Radiologie hinaus erscheint sinnvoll.
- Die Integration künstlicher Intelligenz in den Prozess verspricht weitere vielfältige Vorteile für eine effizientere und individualisierte Versorgung von Patientinnen und Patienten.
